# Risk prediction models for non-invasive ventilation failure in patients with chronic obstructive pulmonary disease: A systematic review

**DOI:** 10.1097/MD.0000000000040588

**Published:** 2024-12-20

**Authors:** Yuming Gao, Bo Yuan, Peng Fan, Mingtao Li, Jiarui Chen

**Affiliations:** a Department of Respiratory and Critical Care Medicine, Siping Central People’s Hospital, Siping, Jilin, China.

**Keywords:** chronic obstructive pulmonary disease, non-invasive ventilation, prediction models

## Abstract

**Background:**

Chronic obstructive pulmonary disease (COPD) is a common respiratory condition with high morbidity and mortality. Noninvasive mechanical ventilation (NIV) is often used to manage acute COPD exacerbations, but failure can lead to worse outcomes. This systematic review aimed to evaluate risk prediction models for NIV failure in patients with COPD.

**Methods:**

PubMed, Embase, Web of Science, The Cochrane Library, CINAHL, CBM, CNKI, Wanfang, and VIP databases, from database inception to January 10, 2024, were searched for studies on risk prediction models for failure in NIV among COPD patients. Two reviewers independently screened the literature, extracted data, assessed the quality of included studies using the Prediction Model Risk of Bias Assessment Tool, and conducted a systematic evaluation of the prediction models.

**Results:**

A total of 11 studies were included, encompassing 13 risk prediction models. The area under the receiver operating characteristic curve for the included models ranged from 0.810 to 0.978. Predictive factors in the models mainly included Acute Physiology And Chronic Health Evaluation II score, pH value, PaCO_2_, consciousness status, serum albumin level, and respiratory rate.

**Conclusion:**

Existing risk prediction models for failure in NIV among patients with COPD demonstrated overall good predictive performance, but exhibited a risk of bias. Further validation is needed to assess the clinical applicability of these models.

## 1. Introduction

Chronic obstructive pulmonary disease (COPD) is a prevalent chronic respiratory system disorder characterized by its high incidence and mortality rate; the disease significantly impacts patients’ work capacity and quality of life.^[1]^ For patients experiencing acute exacerbations of chronic obstructive pulmonary disease or concurrent respiratory failure, noninvasive positive pressure ventilation (NPPV) is considered a crucial therapeutic intervention alongside pharmacological treatment.^[2]^ NPPV effectively enhances pulmonary ventilation function, reduces the need for endotracheal intubation, shortens hospitalization duration, and mitigates the occurrence of complications such as severe respiratory tract damage and ventilator-associated pneumonia.^[3]^ Nevertheless, some patients may experience treatment failure using NPPV, with the failure rate in patients with mild-to-moderate respiratory failure ranging from 20% to 30%, while it ranges from 38% to 62% in those with severe respiratory failure.^[4–6]^ Both domestic and international studies suggest that the failure of noninvasive mechanical ventilation in COPD patients is associated with factors such as acidosis, procalcitonin levels, C-reactive protein, and inadequate relief from or worsening of carbon dioxide retention.^[7,8]^ Some patients may require transition to endotracheal intubation or invasive mechanical ventilation due to NPPV treatment failure that poses a severe threat to their lives. Therefore, accurately identifying patients with suboptimal outcomes from noninvasive mechanical ventilation is crucial for improving rescue success rates and enhancing patient prognosis.

Researchers have conducted studies to explore risk factors associated with NPPV failure in COPD patients and developed risk prediction models based on these factors. However, a systematic evaluation of these models is lacking, and the practicality of each model remains unclear. Hence, this study aims to systematically review the construction methods, presentation formats, predictive performance, and predictive factors of risk prediction models for noninvasive mechanical ventilation failure in COPD patients. The findings are aimed at providing a basis for clinical practitioners in the selection or further development of appropriate predictive models.

## 2. Methods

The study protocol has been registered with PROSPERO (registration number: CRD42023451050).

### 2.1. Search strategy

A comprehensive search was conducted in the PubMed, EMbase, Web of Science, The Cochrane Library, CINAHL, CBM, CNKI, Wanfang, and VIP databases for studies related to risk prediction models for noninvasive mechanical ventilation failure in COPD patients. The search covered the inception of the databases until January 10 2024, and it employed a combination of subject terms and free-text terms. The English search terms included: Chronic Obstructive Lung Disease, COPD, COAD, Chronic Obstructive Airway Disease, Chronic Airflow Obstruction, AECOPD, Acute Exacerbation of Chronic Obstructive Pulmonary Disease, Chronic Airflow Limitation, Noninvasive Ventilation, Non-Invasive Ventilation, Non-Invasive Ventilation, noninvasive Mechanical Ventilation, Failure, Model, Prediction, Prediction Model, Clinical Prediction Model, Risk Prediction, Risk Score, Risk Assessment. The Chinese search terms included: Chronic Obstructive Pulmonary Disease, COPD, Acute Exacerbation of Chronic Obstructive Pulmonary Disease, Noninvasive Mechanical Ventilation, Noninvasive Ventilation, Noninvasive Respiratory Support, Noninvasive Assisted Ventilation, Failure, Model, Prediction Model, Risk Prediction, Risk Score, Forest Plot. We further supplemented our study selection by scrutinizing the bibliographies of the identified studies and review articles to identify additional pertinent research sources.

### 2.2. Inclusion and exclusion criteria

Inclusion criteria: (1) the study types included cohort studies, case–control studies, or cross-sectional studies; (2) the focus of the research was on the construction of risk prediction models for noninvasive mechanical ventilation failure in COPD patients, including detailed model development and validation processes; (3) the study participants were individuals aged ≥ 18 with COPD or those experiencing acute exacerbation of chronic obstructive pulmonary disease (AECOPD); (4) the predicted outcome was noninvasive mechanical ventilation failure; (5) studies available in both Chinese and English were examined.

Exclusion criteria: (1) studies that pertained to risk factor analysis without model construction; (2) studies with fewer than 2 predictive variables; (3) studies that included patients undergoing invasive mechanical ventilation treatment; (4) studies with inaccessible full-text documentation, or where valid data could not be extracted, such as conference abstracts; (5) basic research studies, including animal experiments.

### 2.3. Study selection and screening

The literature retrieved from each database was initially imported into Note Express software for deduplication. Subsequently, the inclusion of literature was determined through the examination of titles, abstracts, and full texts. Two researchers independently screened the literature based on the inclusion and exclusion criteria. Following this, a standardized data extraction form was developed, adhering to the Critical Appraisal and Data Extraction for Systematic Reviews of Prediction Modelling Studies (CHARMS) guidelines.^[9]^

### 2.4. Data extraction

Qualification assessment of the full-text reports was conducted by the 2 reviewers, and any discrepancies were resolved through discussion or adjudication by a third reviewer. Information extracted from the selected studies including details such as author, publication year, study design, country, participants, model forecast results, model development approaches, discrimination AUC, calibration, selection method for predictor variables, internal verification, external verification, sample, candidate variables, predictor, and number of events. Data extraction that was performed by 1 reviewer was verified by the second reviewer to ensure accuracy and consistency.

### 2.5. Risk of bias and assessment of suitability

Two researchers independently assessed the bias risk and applicability of the included models using the Prediction Model Risk of Bias Assessment Tool.^[10]^ Again, cross-checking was performed, and in cases of disagreement, a third party’s input was sought. The bias risk assessment covered 4 domains, viz. study participants, predictors, outcomes, and analysis. These domains comprised a total of 20 questions, each answered using “yes/probably yes,” “no/probably no,” or “no information.” If the bias risk was low across all domains, the overall bias risk was considered low. If the bias risk was high in any 1 domain, the overall bias risk was considered high. If the bias risk was unclear in any 1 domain, the overall bias risk was deemed unclear. Applicability assessment covered 3 domains, viz. study participants, predictors, and outcomes. If applicability was considered good in each domain, the overall applicability was deemed good. If applicability was poor in any 1 domain, the overall applicability was considered poor.

## 3. Results

### 3.1. Study selection

**Figure 1.**
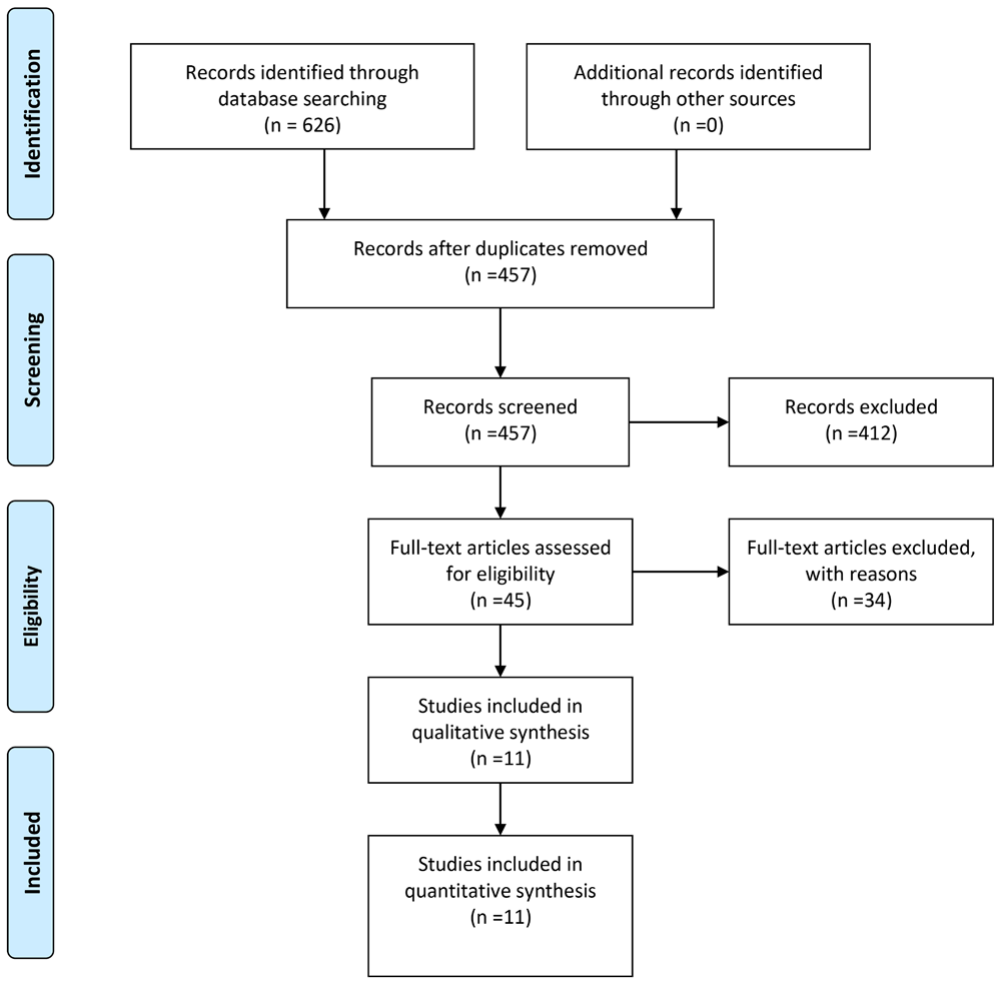
Preferred Reporting Items for Systematic reviews and Meta-Analyses (PRISMA) flowchart of literature search and selection. *Note*: A total of 626 relevant articles were identified through the search. After deleting duplliterature, 457 articles were recorded, and 45 articles were left after evaluating the full text of the literature, and 11 articles were finally included according to the volume exclusion criteria.

A total of 626 relevant articles were identified through the search, and after deduplication and multiple rounds of screening, 11 articles were ultimately included,^[11–21]^ encompassing 13 predictive models. Notably, Confalonieri et al^[13]^ and Duan et al^[16]^ each proposed 2 models. The screening process is illustrated in Figure 1.

### 3.2. Study characteristics

Table 1 provides an overview of the selection and performance of the included models. Among the 11 articles included in this study,^[11–21]^ 5 were in Chinese,^[14,18–21]^ and 6 were published in English.^[11–13,15–17]^ Five studies employed a prospective cohort design,^[11–13,16,17]^ while 6 adopted a retrospective case–control design.^[14,15,18–21]^ The study participants were COPD or AECOPD patients receiving noninvasive mechanical ventilation treatment in hospital settings. “Failure of noninvasive ventilation treatment,” was defined as persistent or worsening symptoms after treatment, potentially requiring endotracheal intubation. However, in 2 reviewed studies,^[14,17]^ the criteria used to determine this outcome were not explicitly reported.

**Table 1:** Basic characteristics of the included literature.

Author	Year	Country	Study design	Participants	Indications of treatment failure	Model development Method/presentation	Selection method for predictor variables	AUC/ Sensitivity/specificity	Calibration	Internal verification	External verification	Sample	Number of events	Candidate variables	Predictor
Antonio Antón et al^[11]^	2000	Spain	Prospective cohort study	Patients with chronic airflow limitation presenting with acute respiratory failure in the respiratory medicine department	Clinical symptoms and pulmonary function variables persisted or worsened	Logistic regression model	Logistic multifactorial variables	None/0.970, 0.900	None	There was internal validation but no details of specific methods	None	34	10	11	pH, PaCO_2_, score of consciousness state
S. Putinati et al^[12]^	2000	Italy	Prospective cohort study	Patients with COPD accompanied by acute respiratory failure treated with NIPPV in the respiratory medicine department.	Required tracheal intubation or death during NIPPV	Logistic regression model	Logistic multifactorial variables	None/0.604, 0.884	None	There was internal validation but no details of specific methods	None	58	17	11	Weight, APACHE Ⅱ score, serum albumin levels
M. Confalonieri et al^[13]^	2005	Italy	Prospective cohort study	Patients with AECOPD who underwent noninvasive ventilation	Tracheal intubation required	Logistic regression/nomographic chart	Logistic multifactorial variables	① The C statistic at admission was 0.810, sensitivity and specificity were 0.330 and 0.967, respectively, and the C statistic was 0.880 sensitivity and specificity were 0.529 and 0.941, respectively	None	Cross-validation	There was external validation but no details of specific methods	1033	236	14	APACHEⅡ score, Glasgow coma GCS score, respiratory rate, pH
Lina Xu et al^[14]^	2011	China	Retrospective study	Patients with AECOPD combined with type II respiratory failure receiving NIPPV.	None	Logistic regression model	Logistic multifactorial variables	ROC: 0.903 No sensitivity, no specificity	Hosmer–Lemeshow test	There was internal validation but no details of specific methods	None	176	40	15	Arterial PaCO_2_ values and Glasgow coma GCS score at 2 hours after noninvasive treatment
Jinrong Wang et al^[15]^	2019	China	Retrospective study	Patients with AECOPD requiring NIV admitted to ICU and referred from general wards	The patient did not improve or even worsened during NIV treatment	Logistic regression/none	Logistic multifactorial variables	0.978, No sensitivity and specificity	None	None	None	376	90	17	Procalcitonin, C-reactive protein, and PaCO_2_
Jun Duan et al^[16]^	2019	China	Prospective cohort study	Patients with COPD undergoing NIPPV in the respiratory ICU	①Intubation or death during the NIV. ② Early NIV failure: NIV failure occurred within 48 hours of NIV	Logistic regression risk-scoring system	Logistic multifactorial variables	① NIV failure: 0.900,0.702,0.926 ② Early NIV failure: 0.910,0.813,0.899	Hosmer–Lemeshow test	There was internal validation but no details of specific methods	There was external validation but no details of specific methods	500	188	16	Heart rate, pH, consciousness (assessed by Glasgow Coma GCS score), oxygenation, respiratory rate
Yang Weng et al^[17]^	2019	China	Prospective cohort study	COPD patients undergoing NIPPV in the respiratory department	None	GPC Bayesian nonparametric prediction model/none	GBDT	0.867, 0.900, 0.950	None	There was internal validation but no details of specific methods	None	144	None	None	Duration of the disease, respiratory rate, PaCO_2_, age, pH, plasma albumin, PaO_2_, gender
Hongrui Zhai et al^[18]^	2020	China	Retrospective study	Patients with AECOPD who underwent noninvasive ventilation	After noninvasive ventilation, there was no improvement or progressive aggravation, and endotracheal intubation was required	Logistic regression model	Logistic multifactorial variables	0.818 ± 0.051, 0.815, 0.680	Hosmer–Lemeshow test	None	None	118	17	26	Respiratory index, pH, white blood cell count, and sputum discharge disorder
Weichao Ding et al^[19]^	2021	China	Retrospective study	Patients with AECOPD who underwent noninvasive ventilation in the EICU	After noninvasive ventilation, there was no improvement or progressive aggravation, and endotracheal intubation was required	Logistic regression/none	Logistic multifactorial variables	0.840, 0.867, 0.812	None	None	None	78	30	21	PaCO_2_, albumin levels
Liu Chun et al^[20]^	2021	China	Retrospective study	Patients with COPD and combined respiratory failure treated with NIPPV	The patient had no improvement or significant deterioration, and new symptoms eventually led to endotracheal intubation or death	Logistic regression/none	Logistic multifactorial variables	0.960, 0.803, 0.913	None	None	None	128	38	21	APACHEⅡ score, pH values after 4 hours of treatment, PaCO_2_
Dawen Zhou et al^[21]^	2023	China	Retrospective study	Patients with COPD and respiratory failure undergoing noninvasive ventilator therapy	The dyspnea did not improve, the oxygenation index was difficult to maintain, and the ventilation treatment was resumed within 48 hours after the withdrawal	R language/nomogram	Logistic multifactorial variables	0.871, No sensitivity, no specificity	Hosmer–Lemeshow test	There was internal validation but no details of specific methods	None	497	129	16	Age, duration of mechanical ventilation, pretreatment PaO_2_, pretreatment PaCO_2_, pretreatment serum albumin, pretreatment CRP, and APACHEⅡ score on admission

AECOPD = acute exacerbations of chronic obstructive pulmonary disease; APACHE = acute physiology and chronic health evaluation; AUC = area under curve; COPD = chronic obstructive pulmonary disease; EICU = emergency intensive care unit; ICU = intensive care unit; GBDT = Gradient Lift Decision Tree; GPC = Gaussian Process Classification; NIPPV = noninvasive positive pressure ventilation; NIV = noninvasive ventilation.

### 3.3. Model development

In the selection of predictive variables, 10 studies^[11–16,18–21]^ utilized logistic multivariable analysis, while 1 study^[17]^ employed a decision tree for variable selection. In terms of model construction, 9 studies^[11–16,18–20]^ applied logistic regression, 1 study^[21]^ utilized an R language package, and another study^[17]^ employed Gaussian Process Classification to construct a Bayesian nonparametric prediction model. Four models^[11,12,14,18]^ derived risk assessment formulas based on logistic regression coefficients or manipulated values according to coefficients. Two models^[13,21]^ presented risk prediction models in the form of forest plots, 1 model^[16]^ presented it in the form of a risk score, and 4 models^[15,17,19,20]^ did not provide a specific presentation. The total sample size ranged from 34 to 1033 cases, with event counts ranging from 10 to 236.

### 3.4. Model performance and predictive factors

Among the 13 models, 11 reported discrimination metrics during model construction and/or validation. The area under the receiver operating characteristic curve (AUC) for these 11 models ranged from 0.810 to 0.978, all exceeding 0.7, indicating good predictive performance. Regarding model validation, 2 models^[13,16]^ were based on both internal and external validation, 6 models^[11,13,15–17,21]^ had internal validation only, and 7 models^[12,15,17–21]^ did not report validation details. The number of candidate variables in the 11 included studies ranged from 11 to 26; the final models comprised 2 to 8 covariates. Common predictive factors included high acute physiology and chronic health evaluation II (APACHE II) score, low PH value, elevated PaCO_2_, impaired consciousness status, low serum albumin levels, and rapid respiratory rate.

### 3.5. Model bias risk and applicability assessment

Specific bias risk and applicability assessments are presented in Table 2. In an analysis of bias risk, 4 domains (participants, predictors, outcome, and statistical analysis) were examined. Nine studies^[11,12,14–16,18–21]^ among the 11 were overall assessed with high bias risk, while the remaining 2 studies^[13,17]^ had unclear bias risk. In the domain of study participants, 6 studies were rated as high risk, while 5 were assessed as low risk. All the 11 studies established strict inclusion and exclusion criteria, but 6 studies^[14,15,18–21]^ were considered high risk in this domain owing to their retrospective nature. In the domain of predictive factors, all those in the prediction models were deemed effective in the 11 studies and were accordingly assessed as low risk in this regard. In the outcome domain, 9 studies were rated as low risk, while 2 studies were assessed as unclear. Here, the models developed by Xu Lina et al^[14]^ and Yang Weng et al^[17]^ did not report the criteria for determining “failure of noninvasive mechanical ventilation.” Hence, the definition was unclear, and so the bias risk was accordingly deemed unclear for these studies. In the statistical analysis domain, 8 studies had a higher risk of bias, and 3 studies were unclear. The main reasons included: (1) 6 studies^[11,12,18–21]^ had insufficient sample sizes with events per variable (EPV) < 20; 1 study did not report the number of event occurrences; (2) only 2 studies^[14,18]^ reported the handling of continuous and categorical independent variables, converting continuous variables into categorical variables with ≥ 2 categories; (3) all studies did not report missing data and handling methods; (4) only 4 studies^[14,16,18,21]^ used the Hosmer–Lemeshow goodness-of-fit test to assess model calibration; (5) 5 studies^[12,15,18–20]^ did not report information on internal validation.

**Table 2:** Risk of model bias and applicability evaluation.

Study	Risk of bias				Applicability			Overall	
	Participants	Predictors	Outcome	Analysis	Participants	Predictors	Outcome	Risk of bias	Applicability
Antonio Antón et al^[11]^	Low	Low	Low	High	Good	Good	Good	High	Good
S. Putinati et al^[12]^	Low	Low	Low	High	Good	Good	Good	High	Good
M. Confalonieri et al^[13]^	Low	Low	Low	Unclear	Good	Good	Good	Unclear	Good
Lina Xu et al^[14]^	High	Low	Unclear	High	Good	Good	Poor	High	Poor
Jinrong Wang et al^[15]^	High	Low	Low	Unclear	Good	Good	Good	High	Good
Jun Duan et al^[16]^	Low	Low	Low	High	Good	Good	Good	High	Good
Yang Weng et al^[17]^	Low	Low	Unclear	Unclear	Good	Good	Poor	Unclear	Poor
Hongrui Zai et al^[18]^	High	Low	Low	High	Good	Good	Good	High	Good
Weichao Ding et al^[19]^	High	Low	Low	High	Good	Good	Good	High	Good
Liu Chun et al^[20]^	High	Low	Low	High	Good	Good	Good	High	Good
Dawen Zhou et al^[21]^	High	Low	Low	High	Good	Good	Good	High	Good

In terms of model applicability, 3 domains (participants, predictors, and outcome) were examined. Two studies^[14,17]^ were assessed as poor due to the lack of reporting outcome definitions, while the other 9 studies had good applicability and were assessed as low risk.

## 4. Discussion

### 4.1. Models exhibit good predictive performance, but overall bias risk is high

This study systematically reviewed relevant literature (from both domestic Chinese and international sources) on risk prediction models for noninvasive mechanical ventilation failure in COPD patients, ultimately including 13 models from 11 studies. The measure of discrimination of these models ranged from 0.810 to 0.978, with 2 studies^[11,12]^ not reporting AUC values. The C-index or AUC of <0.5 indicates poor performance, 0.5 to 0.7 is considered fair, 0.7 to 0.8 is acceptable, 0.8 to 0.9 is excellent, and above 0.9 is considered outstanding.^[22]^ Therefore, the included models demonstrated good predictive performance. However, upon summarizing calibration, validation, and other model reporting aspects, it was found that the quality of model reporting was somewhat lacking, with a high risk of bias and some models requiring improvement in applicability. The lack of calibration and validation for most models is a common issue. Among the included studies, only 4^[14,16,18,21]^ reported model calibration, all using the Hosmer–Lemeshow goodness-of-fit test. Only 2 studies^[13,16]^ performed external validation in addition to internal validation, and 5 studies^[12,15,18–20]^ did not report model validation information, potentially leading to overfitting that could compromise model quality and clinical application. To enhance the quality and clinical applicability of relevant risk prediction models, future research could consider conducting multicenter, large-sample studies nationwide, applying multiple models, and validating them with diverse populations. Strict adherence to the TRIPOD statement for model development, updating, and validation should be observed, with as much information presented as possible about model construction. This will further validate the applicability and safety of these models in clinical practice.

### 4.2. High-risk factors for non-invasive mechanical ventilation failure in COPD patients

The final models included 2 to 8 predictive factors, with commonly assigned and highly weighted factors across various prediction models. These factors primarily included arterial blood pH, PaCO_2_, consciousness, APACHE II score, serum albumin levels, and respiratory rate (Table 1). Arterial blood pH and PaCO_2_ were the most frequently identified high-weighted factors. Arterial blood gas analysis is a crucial method for assessing oxygen status and lung function in the human body. Due to obstructive ventilation impairment and worsening emphysema in COPD patients, severe hypoxia and carbon dioxide retention often occur. Analysis of patients’ arterial blood gas results can effectively evaluate the severity of the condition and prognosis.^[22]^ Multiple studies have indicated that results from pH and arterial blood gas analysis, including PaCO_2_, and PaO_2_ can accurately predict the effectiveness of noninvasive ventilation therapy.^[23–25]^ However, there is some controversy regarding the monitoring time for changes in blood gas analysis indicators. Xu et al’s study^[14]^ measured arterial blood PaCO_2_ values 2 hours after noninvasive treatment, while Chun et al’s^[20]^ chose to measure the reading 4 hours after treatment. Patients in the early stages of noninvasive ventilation therapy need to gradually adapt to improvements in ventilation while also adjusting respiratory machine parameters step by step. This may result in a lack of obvious improvement in arterial blood gas analysis-related indicators. Therefore, further research is needed to validate the appropriate monitoring time. In this study, 4 prediction models included “consciousness level” as a predictive factor, all assessed using the Glasgow Coma Scale. In COPD patients, severe carbon dioxide retention can lead to hypercapnic encephalopathy, further causing hemodynamic instability, respiratory suppression, and worsening consciousness disorders. Patients with impaired consciousness cannot effectively cooperate with the ventilator, often leading to the failure of noninvasive ventilation therapy.^[26]^

APACHE II score^[27]^ is an indicator reflecting the severity of a disease. Relevant studies have indicated that those with better clinical efficacy show a significant decrease in APACHE II scores.^[28]^ A higher score indicates a more severe condition, with a higher failure rate in noninvasive ventilation treatment. Body weight and serum albumin levels are indicators of the nutritional status of COPD patients. Low body weight and serum albumin levels suggest a risk of malnutrition, which may lead to respiratory weakness and cough, decreased resistance, and susceptibility to infections that affect treatment outcomes.^[29]^ In addition, elevated inflammatory markers and sputum clearance disorders are also predictive factors for noninvasive ventilation failure in COPD patients.^[30]^ The inflammatory markers involved in this study mainly include white blood cell count, procalcitonin, and C-reactive protein. Most COPD patients experience symptoms of respiratory tract infections due to the colonization of respiratory pathogens, especially AECOPD patients who require noninvasive ventilation treatment. Lung viral or bacterial infections and substantial colonization aggravate airway inflammation, leading to an increase in white blood cell count, procalcitonin, and C-reactive protein. The result is a poorer patient prognosis.

Sputum clearance disorders and ineffective airway drainage can cause a decrease in effective ventilation, leading to or exacerbating CO_2_ retention. The result is a noninvasive ventilation treatment that is more prone to failure. In the future, further exploration of the mechanisms of noninvasive ventilation treatment failure in COPD patients is necessary to identify specific predictive factors, thereby enhancing the accuracy of predictions and remedial measures. In clinical practice, close monitoring of relevant indicators in COPD patients undergoing noninvasive ventilation treatment is essential for identifying early signs of treatment failure as well as implementing targeted preventive measures. These efforts are aimed at improving patients’ respiratory functions and increasing the success rate of treatment.

### 4.3. Prediction models for NPPV failure in COPD patients are in the initial development stage, and high-quality models require further development and validation

This study included only 1 model^[15]^ based on machine learning algorithms for predicting the risk of noninvasive positive pressure ventilation (NPPV) failure in COPD patients. Among the remaining models, 1^[21]^ utilized the R language to construct a nomogram, while others employed logistic regression for model development. Machine learning algorithms based on medical big data exhibit unique advantages in handling high-dimensional variables, complex interactions between variables, and nonlinear relationships.^[31]^ Therefore, future efforts could leverage data mining, machine learning, and artificial intelligence technologies to develop more precise and predictive models for predicting the risk of NPPV failure in COPD patients. One limitation of the models included in this study is the issue of small sample sizes. When the EPV in model development studies is equal to or >20, the regression coefficients of the model can better approximate the true regression coefficients, resulting in a more accurate regression model. In this study, 4 studies^[13–16]^ had EPV values equal to or >20, 3 studies^[19–21]^ had EPV values between 10 and 20, but 3 studies^[11,12,18]^ had EPV values <10, making the models prone to overfitting. In future developments or external validations of prediction models for NPPV failure in COPD patients, attention should be given to selecting sufficiently large sample sizes to increase EPV so that sampling errors and enhance the reliability of results could be reduced.

With the advancements in diagnostic technologies and medical practices, researchers globally have identified indicators such as diaphragm thickening fraction, transdiaphragmatic pressure,^[32]^ and serum N-terminal pro-B-type natriuretic peptide^[33]^ as having predictive value for NPPV failure in COPD patients. Future research endeavors could consider incorporating these factors into new prediction models to enhance their efficacy.

## 5. Conclusion

This study included a total of 11 articles, encompassing 13 prediction models. Common risk factors for NPPV failure in COPD patients include arterial pH below 7.5, elevated PaCO_2_, impaired consciousness, high APACHE II scores, low serum albumin levels, and rapid respiratory rates. Overall, the predictive performance of the included models is satisfactory, but there is a general tendency toward higher bias risk, indicating a moderate quality of the studies. Future research should focus on further optimizing the model construction process, validation, and result reporting to enhance stability and generalizability. Additionally, improving the clinical applicability of the models, enhancing healthcare professionals’ early recognition of NPPV failure in COPD patients, and timely adoption of personalized follow-up treatment measures are crucial for reducing adverse outcomes in COPD patients.

## 6. Limitations

This study has several limitations: (1) due to variations in data sources and assessment tools, a complete meta-analysis was not feasible, and so only qualitative summaries are provided. (2) Five out of the 11 studies included did not describe model validation, resulting in higher risk of bias. (3) The study only included literature in Chinese and English, predominantly consisting of small-sample, single-center studies that can potentially introduce publication bias.

## Author contributions

**Conceptualization:** Yuming Gao, Bo Yuan, Peng Fan.

**Data curation:** Yuming Gao, Bo Yuan.

**Formal analysis:** Bo Yuan, Mingtao Li, Jiarui Chen.

**Methodology:** Yuming Gao, Bo Yuan.

**Writing – original draft:** Yuming Gao.

**Writing – review & editing:** Yuming Gao.
